# Immobilization of purified pectinase from *Aspergillus nidulans* on chitosan and alginate beads for biotechnological applications

**DOI:** 10.1186/s12934-024-02603-x

**Published:** 2025-01-04

**Authors:** Hamed M. El-Shora, Sabah A. Abo-Elmaaty, Gharieb S. El-Sayyad, Widad M. Al-Bishri, Ahmed I. El-Batal, Mervat G. Hassan

**Affiliations:** 1 Botany Department, Faculty of Science, Mansoura University, Mansoura, Egypt; 2 Botany and Microbiology Department, Faculty of Science, Benha University, Benha, Egypt; 3 Medical Laboratory Technology Department, Faculty of Applied Health Sciences Technology, Badr University in Cairo (BUC), Cairo, Egypt; 4 Microbiology and Immunology Department, Faculty of Pharmacy, Galala University, New Galala City, Suez, Egypt; 5 Drug Microbiology Lab., Drug Radiation Research Department, National Center for Radiation Research and Technology (NCRRT), Egyptian Atomic Energy Authority (EAEA), Cairo, Egypt; 6 Department of Biological Sciences, College of Science, University of Jeddah, 80327 Jeddah, Saudi Arabia

**Keywords:** Pectinase, Orange, Mango, Pineapple, Chitosan, Alginate, Antioxidant activity

## Abstract

**Background:**

Because the process is cost-effective, microbial pectinase is used in juice clearing. The isolation, immobilization, and characterization of pectinase from *Aspergillus nidulans* (Eidam) G. Winter (AUMC No. 7147) were therefore the focus of the current investigation.

**Results:**

Ammonium sulphate (85%), DEAE-cellulose, and Sephadex G-200 were used to purify the enzyme. With a yield of 30.4%, the final specific activity was 400 units mg^−1^ protein and 125-fold purification. Using SDS-PAGE to validate the purification of the pectinase, a single band showing the homogeneity of the purified pectinase with a molecular weight of 50 kD was found. Chitosan and calcium alginate both effectively immobilized pectinase, with immobilization efficiencies of 85.7 and 69.4%, respectively. At 50, 55, 60, and 65 °C, the thermostability of both free and chitosan-immobilized pectinase was examined. The free and chitosan-immobilized enzymes had half-lives (t_1/2_) of 23.83 and 28.64 min at 65 °C, and their K_d_ values were 0.0291 and 0.0242 min^−1^, respectively. In addition, the Z values were 44.6 and 31.54 °C, while the D values were 79.2 and 95.1 min. Compared to the untreated one, the orange, mango, and pineapple juices treated with immobilized pure pectinase showed greater clarity. Following treatment with pure pectinase, the fruit juice’s 1, 1-diphenyl-2-picrylhydrazyl and 2, 2′-azino-bis 3-ethylbenzothiazoline-6-sulfonate scavenging activities increased. Following treatment with pure pectinase, the amounts of total phenolics and total flavonoids increased.

**Conclusion:**

The procedure is deemed cost-effective in the food industry because the strong affinity of fungal pectinase for pectin. The investigated pectinase supported its usage in the food industry by being able to clear orange, mango, and pineapple juices.

## Background

Higher plants’ middle lamella and major cell walls contain pectin in a calcium and magnesium pectate combination [16]. Pectins, pectinic acids, protopectins, and/or polygalacturonic acids are the primary components of heterogenic pectic contents [3, 26]. Other sugars including d-mannose, l-fucose, d-glucuronic acid, d-glucose, and d-xylose are reported to be present in the side chains of compounds that include pectin [55].

Microbial enzymes are employed in a variety of industrial contexts due to their well-established role as metabolic catalysts. According to Arora et al. [5], industrial enzymes have a very large end-use market with an extensive spectrum of industrial and commercial purposes. Microbes have been and continue to be one of the most plentiful and advantageous sources of a number of enzymes [63].

Numerous microorganisms, including yeast [41], fungi [69], bacteria [34], and streptomyces [58], generate pectinase enzymes. Most readily accessible microbial pectinase is derived from fungal sources, primarily *Aspergillus* species [33]. Approximately 25% of industrial enzyme sales globally are attributed to the pectinase enzyme [24, 60].

The increasing demand for pectinase has eliminated the need to find microbial strains capable of producing new pectinases with improved activity [35]. Based on how they function on the substrate, pectinase enzymes are categorized as pectinesterase (PE), polygalcturonase (PG), and pectin lyase (PL) [72].

Pectinases are commonly utilized in waste-water management [65], and in the food sector [20]. To produce bioethanol from lignocellulosic biomass, pectinase and cellulase enzymes have been combined [39]. Because polysaccharides including starch, pectin, cellulose, hemicelluloses, and bonded lignin are present in fruit juices produced via simple extraction, they are turbid, viscous, and foggy.

The turbid fruit juice has a low yield and acceptability and is difficult to concentrate and pasteurize [44]. Industrial pectinases are used to improve product quality and boost fruit juice yield and clarity [34]. Several restrictions apply to the industrial application of this soluble form of the enzyme: unstable, uncontrolled recovery and reuse, limited shelf life, handling challenges, and loss of activity under extended conditions of operation [80]. Thus, immobilization can improve these biocatalysts’ affinity, pH stability, functionality stability, and thermostability [49].

Because of its special qualities, including its abundance of function groups, accessibility, biodegradability, and chemical resistance, chitosan is one of the most often utilized support materials for enzyme immobilization [81]. The chitosan structure’s amino group content permits crosslinking modification as well. The most common crosslinking agent that can improve the stiffness, thermal endurance, and the ability to absorb of chitosan backing is glutaraldehyde [37]. Enzyme covalent immobilization on chitosan is achieved by subjecting it to a glutaraldehyde cross-linking solution. The –NH_2_ groups in chitosan and the enzyme react with the dual-functional –CHO groups of glutaraldehyde [61].

Industrial enzymes may be derived from the filamentous fungus *Aspergillus nidulans*, and a variety of industrial enzymes may be produced by this versatile fungal cell factory [45].

Therefore, the current study’s original objective was to discover and immobilize *Aspergillus nidulans* pectinase. Comparing the stability and kinetics of storage for free and immobilized pectinase is the second goal. Thirdly, to determine if free and immobilized pectinase may be used to clarify orange, mango, and pineapple juices.

## Materials and methods

### Experimental microorganism

The Assiut University Mubasher Mycological Center (AUMMC), Assiut, Egypt, is where *Aspergillus nidulans* (Eidam) G. Winter (AUMC No. 7147) was acquired.

### Production of pectinase enzyme

Bhardwaj and Fairhurst [11] state that, the alteration in pectin glucose liquid medium was used for pectinase synthesis. About 100 mL of sterilized medium (g/L) was used in triplicate sets of 250-mL Erlenmeyer flasks having the following concentrations: pectin 5.0, glucose 10, KH_2_PO_4_ 1.0, MgCl_2_·6H_2_O 0.5, NaNO_3_ 1.0, CaCl_2_·2H_2_O 0.1, FeCl_3_·6H_2_O 0.02 and ZnCl_2_ 0.02. After inoculating the medium with a suspension of 1 × 10^7^ spores mL^−1^, the medium was modified to pH 6.0 and cultured for 7 days at 30 °C with 200 rpm of agitation. A crude enzyme was made from the supernatant after the fungal mycelium had been filtered and separated after seven days using centrifugation at 5000×*g* for 5 min.

### Pectinase assay

Miller’s [52] method of assaying the pure pectinase’s activity was followed. About 1 mL of 0.5% pectin, 0.5 mL of sodium acetate buffer (100 mM, pH 5.0), and 0.5 mL of enzyme were included in the test mixture. For 10 min, the reaction combination was incubated in a water bath at 30°°C. The 3, 5-dinitrosalicylic acid (DNS) reagent was added and heated for 15 min after 10 min. Following cooling, the absorbance at a wavelength of 575 nm was measured spectrophotometrically. Galacturonic acid was used to create the standard curve for reducing sugar, and it was intended such that one unit of pectinase would be the amount of enzyme required to generate one µmole of galacturonic acid per minute under reaction circumstances.

### Purification of pectinase

The supernatant containing pectinase was gathered after centrifugation at 5000 rpm for 25 min and mixed with 85% solid ammonium sulphate at 4 °C with permanent stirring and protein precipitation overnight. The precipitate was suspended in 15 mL of 100 mM Tris–HCl buffer, pH 7.5. The resulting pectinase solution was dialyzed versus the same buffer for 24 h with numerous changes to eliminate the salt followed by assaying of pectinase activity as mentioned above.

The obtained fraction from ammonium sulfate fractionation after dialysis was loaded into DEAE-cellulose column (1.5 × 30 cm) as anion exchange chromatography which was pre-equilibrated with the same buffer followed by gradient elution using 100 mM Tris–HCl buffer containing 1 M NaCl at flow rate of 1 mL/min.

The pooled fractions from DEAE-cellulose column with the highest specific activity were added to Sephadex G-200 column (1.5 × 30 cm) as gel filtration chromatography that was pre-equilibrated with 50 mM Tris–HCl buffer, pH 7.5. The same buffer was used for the elution procedure, with a flow rate of one milliliter per minute. The collected pectinase fractions were combined at 4 °C, and using BSA as a reference [13] was followed to calculate the protein content. Units of the enzyme per milligram of protein were used to express the particular activity of pectinase.

### Determination of pectinase molecular weight

Using a wide spectrum of protein markers and the technique of sodium dodecyl sulfate–polyacrylamide gel electrophoresis (SDS-PAGE) (Sigma USA), the molecular weight of pectinase was ascertained using Laemmli’s [47] approach. Phosphorylase B (97 kDa), ovalbumin (50 kDa), carbonic anhydrase (29 kDa), soybean trypsin inhibitor (20 kDa), and lysozyme (14 kDa) were the protein markers. The brilliant blue dye Coomassie was used to see protein bands.

### Immobilization of purified pectinase on chitosan beads

Following El-Shora et al.’s [28] instructions, the cross-linking was completed. About 99 mL of distilled water, three grams of chitosan powder, and 1.5% (v/v) acetic acid were added. The mixture was heated to 60–70 °C, stirred, and left to stand at the ambient temperature for 4 h. After filtering, the mixture was dried. The acetic acid in the chitosan bead was neutralized by adding drops of 2% (w/v) NaOH. After again being cleaned with distilled water, the bead was dried once more.

For 2 h, 1% (w/v) of the dried chitosan bead was added to the glutaraldehyde solution in 100 mM cold phosphate buffer (pH 8.0). Using the same buffer, the brownish reinforced bead was twice cleaned to get rid of any remaining glutaraldehyde. For 4 h, with careful stirring, chitosan beads were combined with two milligrams per milliliter of pectinase liquid in 100 mM phosphate buffer (pH 8.0). By subtracting the activity measured in the supernatant from the total activity supplied to the chitosan beads, the enzyme activity of encapsulated pectinase was assessed. For the enzyme experiment, around 0.1 g of the immobilized pectinase was used.

$${\text{The immobilization efficiency }}(\% ) \, = \, ({\text{specific activity of immobilized enzyme}}/{\text{specific activity of soluble enzyme added}}) \, \times { 1}00.$$

### Immobilization of purified pectinase on alginate bead

The immobilization technique was first taken from El-Shora et al. [30]. After being made in 0.1 M Tris–acetate buffer (pH 8.0) at 70 °C with constant stirring, the sticky solution of sodium alginate (5% w/v) was allowed to cool to 4 °C. There was a mixture of 50 mL sodium alginate (5% w/v) and pure pectinase. The final blend was poured into a separating funnel set over a beaker filled with 150 mL of 4% (w/v) calcium chloride. Next, the alginate bead was arranged by gradually dropping the alginate solution (30 drops min^−1^) into the calcium chloride solution using a 200 μL Eppendorf tip. The bead was gently stirred and left to solidify for 4 h. After the bead was removed from the calcium chloride solution and cleaned with the same buffer, the immobilized pectinase activity was calculated.

### Effect of protein loading on immobilization efficiency

The effect of enzyme loading on immobilized efficiency of pectinase was studied by varying the amount of pectinase offered (1–10 mg g^−1^ bead) to a fixed amount of chitosan or alginate and the immobilization efficiency was calculated.

### Reusability of immobilized pectinase

One of the most crucial characteristics of an immobilized enzyme is its ability to be reused. For this reason, immobile pectinase on triggered beads was utilized repeatedly. Following each reaction, the enzyme-containing beads were thoroughly cleaned using a buffer, reassessed, and the first activity level was recorded as 100%. A percentage of the initial operating activity was used to indicate the relative activity.

### Storage stability of free and immobilized pectinase on chitosan and alginate

The activity was measured over the course of 30 days with the native pectinase, alginate-immobilized pectinase, and chitosan-immobilized pectinase kept at room temperature (25 °C). Every 5 days, samples of the trapped beads (0.1 g) or free pectinase (0.1 mL) were taken out to measure the pectinase activity.

### Thermostability of free and chitosan-immobilized pectinase

Thermostability of free and chitosan-immobilized pectinase was investigated by pre-incubation of each form of pectinase at 50, 55, 60 and 65 °C without substrate. The residual pectinase activity was determined after 10, 20, 30, 40, 50 and 60 min for each tested temperature and expressed as % residual activity.

### Application of chitosan-immobilized pectinase in fruit juice clarification

#### Preparation of fruit juice

We bought fresh orange, mango, and pineapple juices from Mansoura City local market. To lessen potential microbial contamination, deionized water was used to wash the fruits. After cutting a horizontal incision with a knife that was one centimeter deep, the dense fruit skin was carefully peeled to reveal the delicious segments. The fruits were then divided into pieces, and each segment’s inner skin was scraped and thrown away. The juicy sections, containing the seeds, were fully free of the white membrane that surrounded them. A screw-style extractor was used to extract the juice, and nylon filtration was used to get rid of the pulp.

To maximize the extraction of juice, this procedure was carried out three times. For a short while, the freshly squeezed water was pasteurized at 90 °C. To make things clear, the juice’s pHs were raised to pH 8.0 for chitosan-pectinase and pH 7.0 for free pectinase. Centrifugation was performed for 10 min at 10,000 rpm to ensure that all of the juice from every fruit was completely separated.

#### Treatment of juice with crude, soluble purified and chitosan-immobilized enzymes

Since chitosan-immobilized pectinase expressed the highest immobilized efficiency compared to calcium alginate, it was decided to compare the potentiality of crude, soluble purified and chitosan-immobilized enzyme in clarification of fruits juice from orange, mango and pineapple. Two mL from prepared juice of orange, mango and pineapple fruits was mixed individually with 5 mL of crude, soluble purified and 5 mg of chitosan-immobilized pectinase. For 60 min, the process of clarity took place out by incubation at ideal conditions. Juice clarity was assessed upon incubation by using a spectrophotometer to measure the percent transmittance (%T) at 660 nm [6].

#### Determination of total phenol of fruits juice treated with chitosan-immobilized pectinase

Using the Folin-Ciocalteu technique, the total phenol content was determined in the various juices treated with chitosan-immobilized pectinase, as stated by Srinivasan et al. [78]. Using samples (2 mL) of each juice, 5 mL of 0.3% HCl was combined with 100 µL of the mixture, followed by 5 mL of 5% aqueous Na_2_CO_3_, and the mixture was allowed to sit for 10 min. After mixing the mixture with 100 µL of 50% Folin-Ciocalteu’s reagent, it was incubated for 25 min, and the absorbance was measured at 750 nm. To calculate the total phenol content, a typical calibration curve of gallic acid was created and represented in milligrams of gallic acid equivalents (GAEs) per gram of fruit juice.

#### Determination of total flavonoids of fruits juice treated with chitosan-immobilized pectinase

Using the AlCl_3_ technique, the juices’ total flavonoid content was ascertained [51]. Using a vigorous shaker, samples (2 mL) of each juice were collected and combined with 0.1 mL AlCl_3_ (10% w/v), 0.1 mL Na–K tartarate, and 2.8 mL distilled water. After 25 min, the absorbance at 415 nm was read. The total flavonoid, which is reported as mg of the corresponding quercetin per g of material, was calculated using an accepted curve for calibration of quercetin that was generated.

#### DPPH scavenging activity of fruits juice treated with chitosan-immobilized pectinase

The free radical scavenging activity of each prepared juice was determined using 1, 1-diphenyl-2-picrylhydrazyl. Two milliliters of a 0.2 mM methanolic solution of DPPH radicals were combined with two milliliters of the prepared aqueous juice from each fruit. After shaking the mixture and letting it sit in the dark for 25 min, the absorbance at 517 nm was measured [74].

$${\text{DPPH scavenging activity }}\left( \% \right) \, = \, \left[ {\left( {{\text{A}}_{{\text{o}}} - {\text{A}}_{{1}} } \right)/{\text{A}}_{{\text{o}}} } \right] \, \times { 1}00,$$

where A_o_ and A_1_ are the absorbance values in the absence and presence of the test sample, respectively.

#### ABTS scavenging activity of fruits juice treated with chitosan-immobilized pectinase

The aqueous juice of each fruit was tested for its ability to scavenge 2, 2′-azino-bis (3-ethylbenzothiazoline-6-sulfonate, or ABTS) in accordance with Re et al. [64]. In order to create the radical cation (ABTS^•+^), a specific volume of 7.4 mM ABTS was added to 2.6 mM potassium persulphate. The mixture was then left to react for 10–12 h at room temperature in the dark. Following a 10-min room temperature incubation period, 0.5 mL of fruit juice and 3 mL of ABTS^•+^ solution were combined, and the absorbance at a wavelength of 734 nm was measured. The antioxidant activity was calculated by using the following equation:

$${\text{BTS scavenging activity }}\left( \% \right) \, = \, \left[ {\left( {{\text{A}}_{{\text{o}}} - {\text{A}}_{{1}} } \right)/{\text{A}}_{{\text{o}}} } \right] \, \times { 1}00,$$

where A_o_ and A_1_ are the absorbance values in the absence and presence of the test sample, respectively.

### Statistical analysis

ANOVA was employed in the ONE-WAY report to statistically analyze the selections at *P* = 0.05. The data and conclusions were analyzed and evaluated via SPSS software (version 15). In this investigation, every data point was collected in triplicate ± standard error.

## Results and discussion

The fact that enzymes function best under moderate reactions is not advantageous. Enzymes that function under a variety of reaction settings are of interest for industrial applications. Key characteristics of enzymes, such as stability, specific activity, and substrate specificity, can be enhanced chemically [57].

### Purification of pectinase from *Aspergillus nidulans*

**Fig. 1:**
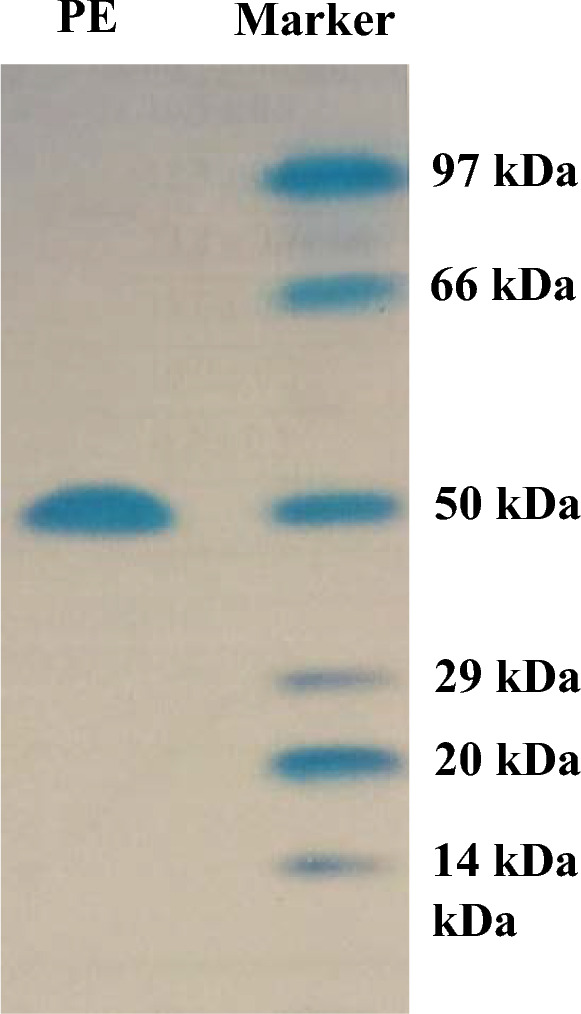
SDS-PAGE of purified pectinase from *A. nidulans*. *PE* pure enzyme

These findings show that the purification procedure was carried out effectively to get a significant 125-fold and 400 U/mg protein specific activity (Table 1). From the literature, *Bacillus subtilis* was used to produce pectinase, which had an 11.6-fold purity and a particular activity of 99.6 U/mg [3]. Moreover, a single band with a molecular weight of 50 kDa for pure pectinase on SDS-PAGE demonstrated the uniformity of the isolated enzyme (Fig. 1).

**Table 1:** Purification of pectinase from *Aspergillus nidulans*

Purification step	Total protein (mg)	Total activity (U)	Specific activity (Umg^−1^ protein)	Yield (%)	Fold of purification
Crude extract	122	395	3.2	100	1.0
85% (NH_4_)_2_ SO_4_	54.0	280	5.0	70.9	1.6
DEAE-cellulose	1.3	160	123.1	40.5	38.5
Sephadex G-200	0.3	120	400.0	30.4	125

According to Khatri et al. [43], pectinase from *Aspergillus niger* showed the last particular activity of 60 U/mg protein with a purification fold of 84 and a yield of 16%. Likewise, pectinase from *Aspergillus tubingensis* [42], *Fusarium oxysporum* [23], *Cochliobolus carbonum* [70], and *Penicillium frequentans* [7] was shown to have a molecular weight of 78, 74, 60, and 63 kDa, respectively.

### Immobilization of pectinase from *Aspergillus nidulans*

Immobilization of pectinase on alginate and chitosan was carried out and the results are shown in Table 2. The immobilized enzyme on chitosan expressed higher immobilization efficiency (85.7%) compared to alginate-immobilized pectinase (69.4%). Immobilization of the enzyme on chitosan is supposed to preserve tertiary structure of enzyme from conformational changes [1]. Each time an enzyme is immobilized, its stiffness increases. This is demonstrated by the increased stability when denaturation occurs at temperatures above the optimum [50]. Chitosan has the advantages of biodegradation, non-antigenicity, good biocompatibility, low cost, non-toxicity and abundant resources. So, chitosan is encouraging organic compound for enzyme immobilization [66]. Alginate is a kind of polysaccharide usually taken from marine algae and alginate structure comprises l-guluronic acid (G) and d-mannuronic acid (M). There are *β* (1–4) glycosidic bonds between d-mannuronic acid molecules and *α* (1–4) glycosidic bond between l-guluronic acid molecules. The molecular structure of alginate may differ depending on the source of organism [79].

**Table 2:** Immobilization efficiency of pectinase from *Aspergillus nidulans* by entrapment and cross-linking methods

Immobilized method	Loaded activity (U mg^−1^ protein)	Immobilized activity (U mg^−1^ protein)	Immobilization efficiency (%)
Cross-linking	41.0 ± 0.9	35.2 ± 0.9	85.7 ± 1.5
Entrapment	41.0 ± 0.9	25.2 ± 0.9	69.4 ± 1.1

The immobilized enzyme offers enhanced resistance against alteration in pH or temperature. In this instance, the enzyme stays in its position the whole reaction, facilitating an easy separation from the result. As a result, immobilization is a useful method for enzyme-catalyzed reactions in industry [73].

### Effect of protein loading on immobilization efficiency of pectinase

**Fig. 2:**
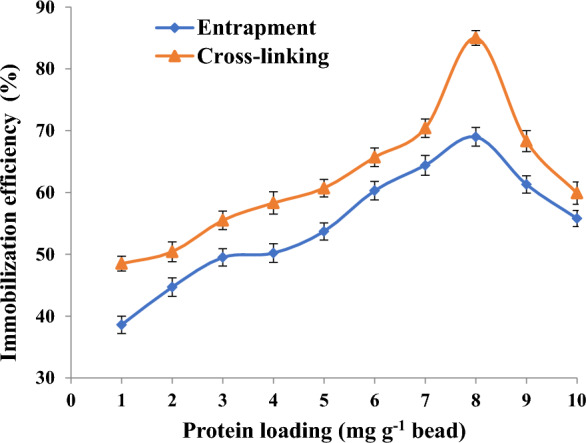
Effect of protein loading on immobilization efficiency of pectinase

As the protein load was increased, the amount of immobilized pectinase increased (Fig. 2). Lower little values of immobilization efficiency are related to too little amounts of enzyme for the quantity of the support used. However, the carrier reached the protein saturation point at a protein amount of 8 mg/g where the immobilization efficiency was 69 and 85%, respectively for alginate and chitosan, respectively. So, at that point, the amount of immobilized protein starts to decline and adding a higher amount of pectinase did not increase the amount of the bound enzyme. The decrease in immobilization efficacy with greater protein loading can be explained by the possibility that excessive protein loading will induce the enzyme to clump together on the support [32, 84].

### Reusability of immobilized pectinase on alginate and chitosan beads

One of the main advantages of immobilized enzymes is their simple process of extraction and reusability. One crucial characteristic for assessing the importance of an enzyme that has been immobilized is its reusability across seven consecutive cycles [29].

**Fig. 3:**
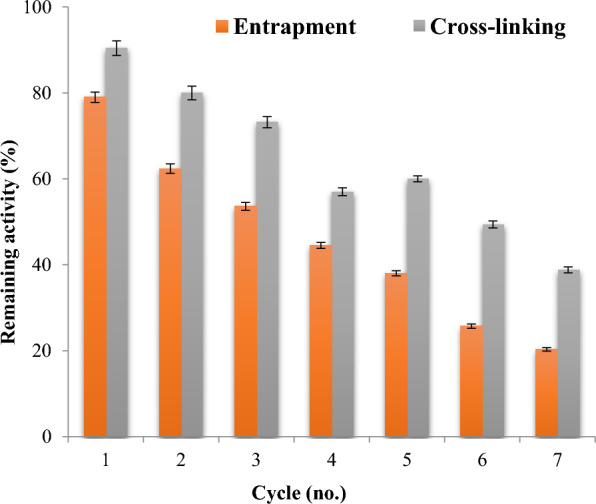
Reusability of entrapped and cross-linked pectinase

It was investigated if the enzymes pectinase and chitosan immobilized in alginate might be reused. The findings, which are displayed in Fig. 3, reveal that after the seventh cycle, the immobilized pectinase maintained 20.3 and 38.8% on alginate and chitosan, respectively.

It is normal for immobilized pectinase to lose activity after repeated usage [77], and may be due to the hydrophilic characteristics of alginate, weak binding via non-covalent bonds, inhibition of pectinase by increasing quinonoid products [53], protein damage, protein deactivation [4] or leakage of the enzyme from support [25]. Immobilized pectinase with good reusability can cut down on the quantity of enzyme utilized in industrial applications, which lowers production costs.

### Storage stability of free and immobilized pectinase at room temperature

**Fig. 4:**
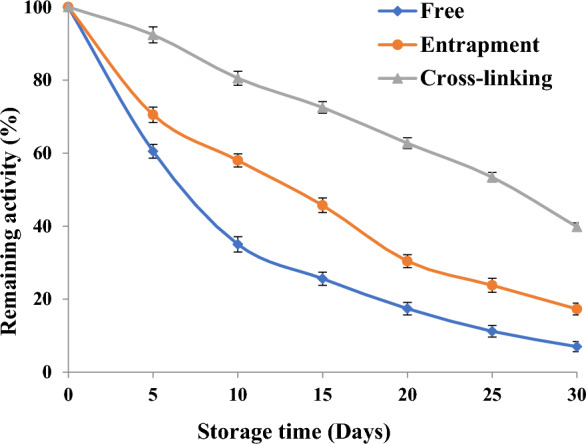
Storage stability of free, entrapped and cross-linked pectinase

Over the course of 30 days at 25 °C, the storage stability of both free and immobilized pectinase was assessed. The residual activity was represented as the original immobilized enzyme’s relative activity. As the storage duration increases, pectinase activity continuously decreases, according to the data in Fig. 4.

Compared to the free enzyme (5%), the immobilized pectinase exhibited greater activity after 30 days, either on chitosan (39.8%) or alginate (17.3%). Because immobilization prevents autolysis, the immobilized pectinase likely displayed greater stability. One possible explanation for the durability of the immobilized pectinase is that the bead provides an environment that is conducive to the enzyme’s growth [27].

These findings show that immobilizing pectinase reduces the likelihood of its denaturation [85], and are consistent with earlier research regarding additional immobilized enzymes [54]. Therefore, it would seem that free pectinase is not robust in storage and that it gradually loses or reduces in activity over time.

### Thermostability of free and chitosan-immobilized pectinase

Enhancing the thermal stability of pectinase to make it suitable for commercial application was one of the primary goals of the current investigation. For both the free and immobilized enzyme, pectinase performed best at temperatures between 40 and 45 °C. Therefore, the thermostability of the free and immobilized pectinase was investigated at 50, 55, 60 and 65 °C and the calculated values of t_1/2_, K_d_ and D at each tested temperature are listed in Table 3.

**Table 3:** The values of half life (t_1/2_), K_d_ and D of free and immobilized pectinase

Temp (°C)	Equation	R^2^	(t_1/2_) (min)	K_d_ (min^−1^)	D (min)
Free pectinase					
50	y = −0.9649x + 100	0.9884	52.8	0.0131	175.4
55	y = −1.3362x + 100	0.996	37.42	0.0185	124.3
60	y = −1.6286x + 100	0.9968	30.70	0.0226	102.0
65	y = −2.0982x + 100	0.9902	23.83	0.0291	79.2
Immobilized pectinase					
50	y = −0.573x + 100	0.9798	87.26	0.0079	289.9
55	y = −1.0047x + 100	0.9907	49.77	0.0139	165.3
60	y = −1.3601x + 100	0.9967	36.76	0.0189	122.1
65	y = −1.746x + 100	0.9804	28.64	0.0242	95.1

**Fig. 5:**
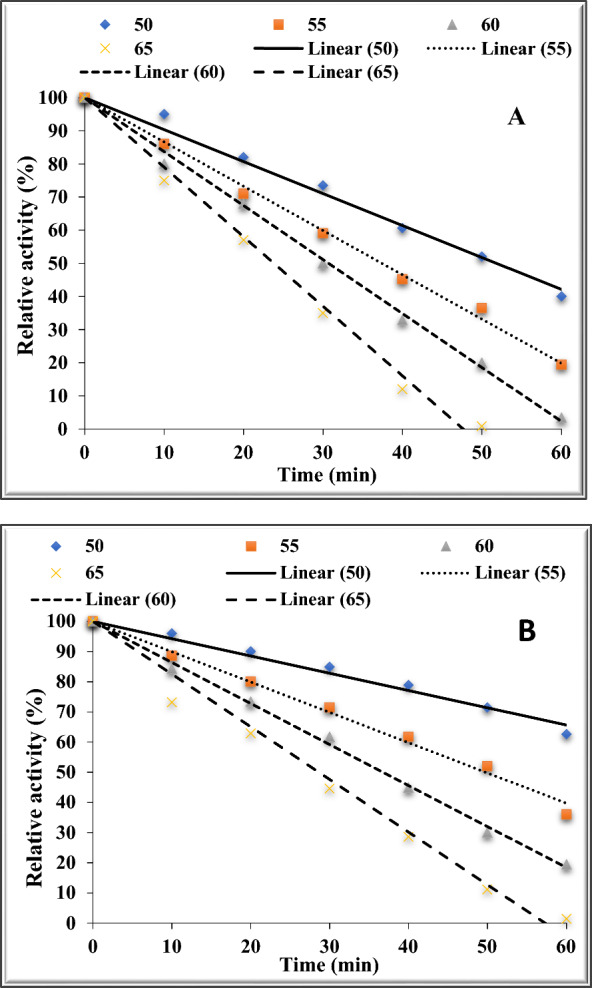
Thermostability of free (**A**) and chitosan-immobilized pectinase (**B**) at 50, 55, 60 and 65 °C

The thermostability findings showed that when the incubation duration was increased at the different investigated temperatures over the ideal one, the activity of free (Fig. 5a), and the immobilized (Fig. 5b) pectinase decreased. On the other hand, compared to the free form, the immobilized pectinase showed greater thermostability.

Pectinase activity from *Penicillium italicum* [2] and *A. fumigates* [59] decreased similarly. Pectinase may become inactive at high temperatures above the optimal temperature because of peptide chain hydrolysis, aggregation, inaccurate confirmation, or amino acid degradation [21]. Pectinase may become inactivated at high temperatures due to build up at hydrophobic sites that become visible during decomposition [82].

It’s still unclear exactly how heat inactivates an enzyme protein, and the incomplete expanding of the enzyme structure is the initial step in the heat inactivation process. Under typical circumstances, the equilibrium between various monovalent ionic forces, such as hydrogen and hydrophobic contact, preserves the catalytically productive structure of the enzyme [71]. The natural three-dimensional arrangement of the enzyme is the only way that multiple amino acid residues are typically assembled to form the sites of activity of the enzyme. The enzyme becomes inactive as a result of this unfolding, which causes the active core to disassemble [18].

Serra [71] states that a variety of modifications take place during thermal inactivation, including non-covalent alterations that allow the thermally unfolded enzyme molecules to shift and covalent changes such the hydrolytic scission of disulfide.

Plotting the residual pectinase activity versus time yielded a slope that was represented as half-life (t_1/2_). The duration required for an enzyme to degrade its substrate and lose half of its activity is known as its half-life (t_1/2_) [9]. The time needed to decrease 50% of the starting enzyme activity at a specific temperature is another way to define the half-life (t_1/2_) [56]. The free pectinase (Fig. 5a and Table 3) had a half-life (t_1/2_) of 23.83 min at 65 °C, whereas the immobilized pectinase (Fig. 5b and Table 3) had a half-life (t_1/2_) of 28.64 min.

According to Lopes et al. [48], the half-life (t_1/2_) was utilized to compute the heat inactivation rate constant (K_d_). The enzyme immobilized in chitosan had a K_d_ value of 0.0242 min^−1^ while the free pectinase had a K_d_ value of 0.0291.

According to Cavalcante Braga et al. [14], deactivation is the process by which a protein’s secondary, tertiary, or quaternary structure changes without any covalent connections being broken. The values given for the two parameters D and Z are typically used to indicate the deactivation of the enzyme [17]. The amount of time the enzyme has to be pre-incubated at a specific temperature in order to retain 10% residual activity is indicated by the D, or decimal reduction value (in minutes) [14].

The time required for a 90% reduction in the beginning activity is another name for the D-value (decimal reduction time), and it was computed in accordance with Singh and Wadhwa [75]. The D values at 65 °C were 95.1 min for the chitosan-immobilized pectinase (Fig. 4 and Table 3) and 79.2 for the free pectinase (Fig. 4 and Table 3). Higher temperatures increased the inactivation rate, but they also decreased the half-life (t_1/2_) and D values, suggesting a quicker rate of deactivation at higher temperatures. Numerous variables, such as pH and the buffer’s structure during thermal inactivation, may be to blame for these discrepancies [56].

**Fig. 6:**
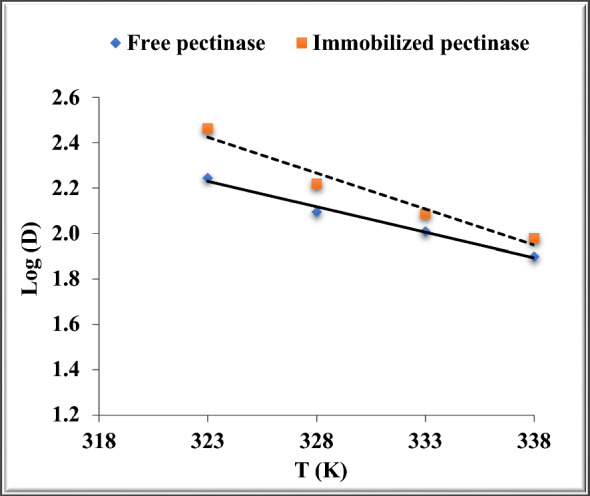
Determination of Z for free and chitosan-immobilized pectinase

The Z-value expresses how the D-value depends on temperature, and log D-values vs. temperature was used to calculate the Z-value. Z values for free and chitosan-immobilized pectinase were 44.6 and 31.54 °C, respectively (Fig. 6 and Table 4). According to Ortega et al. [56], the Z-value shows what number of degrees of temperature is needed to cause a tenfold change in decimal reduction time**.**

**Table 4:** The values of Z for the free and immobilized pectinase

Enzyme form	Equation	R^2^	Z (^o^C)
Free	y = −0.0224x + 9.4811	0.9879	44.6
Immobilized	y = −0.0317x + 12.651	0.9618	31.54

The minimal energy needed to initiate the enzyme’s deactivation process is known as the kinetic energy of deactivation [10]. The linear regression of In(k_d_) vs. reciprocal temperature (1/T) yields the deactivation energy (E_d_) [56].

**Fig. 7:**
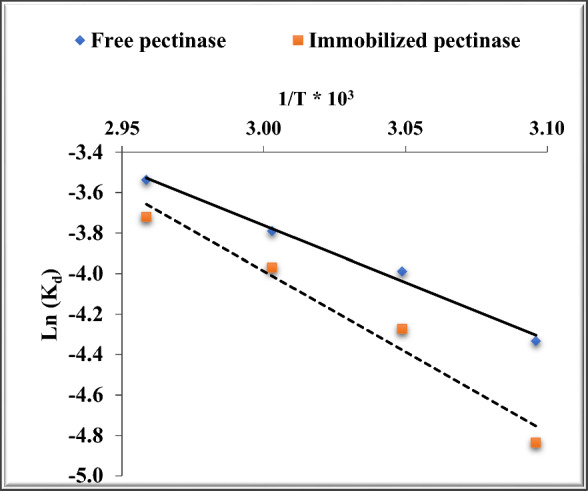
Determination of E_d_ for free and chitosan-immobilized pectinase

Figure 7 and Table 5 show a slight rise in E_d_ following the immobilization treatment. It is important to note that industrial enzyme applications strongly require high Ed values due to their elevated thermostability [31].

**Table 5:** The values of Ed for the free and chitosan-immobilized pectinase

Enzyme form	Equation	R^2^	Ed (kJ/mol)
Free	y = −5.6488x + 13.184	0.9898	46.964
Immobilized	y = −7.9789x + 19.949	0.9666	66.337

### Clarification of juice by crude, soluble purified and chitosan-immobilized purified pectinase

**Fig. 8:**
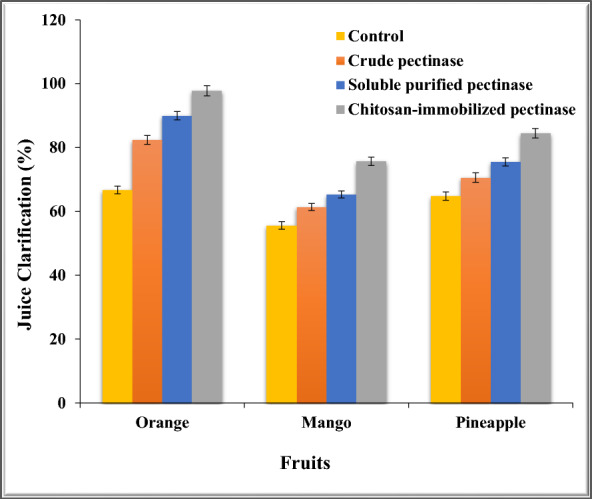
Clarification of various fruit juices by crude, soluble purified and chitosan-immobilized pectinase

The clarification of the various tested fruit juices by crude, soluble purified and chitosan-immobilized purified pectinase was investigated (Fig. 8). The chitosan-immobilized purified pectinase exhibited high clarification (97.8, 75.7 and 84.5%) expressed as juice clarification (%) than the crude enzyme (82.4, 61.4 and 70.6%), soluble purified (90, 65.3 and 75.5%) and the control samples (66.7, 55.6 and 64.8%) for orange, mango and pineapple juices. Juice clarity is achieved by the microbial pectinase’s removal of methyl groups from the pectin backbone. Subsequently, the pectin’s negatively charged areas combine with Ca^2+^ to generate Ca^2+^ pectate gels, which help to clarify juice [46]. Relative with other microorganisms, the pectinase derived from *A. nidulans* demonstrated efficient clarity, suggesting that it might be a viable option for industrial juice clearing. The degree of clarity was enhanced by the fungal pectinase derived from *A. niger* [68], *A. awamori* [22], and *P. oxalium* [83].

### Total phenolics and total flavonoids of fruits juice treated with chitosan-immobilized pectinase

According to reports, phenolics have drawn attention because of their potential for therapeutic use, particularly in the areas of anti-inflammatory, anti-cancer, hypolipidemic, and hypoglycemic fields [15]. Also, fruits are rich in nutrients and contain a variety of phenolics. The phenolic compounds are described as molecules comprising at least one benzene ring to which one or more hydroxyl groups are connected.

An important fraction of the phenolic compounds is connected with a variety of flavor properties, especially astringency. Phenolic compounds found in the plants-based foods are divvied into two major groups: flavonoids and phenolic acids [12]. Since chitosan-immobilized pectinase exhibited higher clarification of the tested fruits it was decided to determine the total phenols and total flavonoids in the various juices treated with this form of pectinase.

**Fig. 9:**
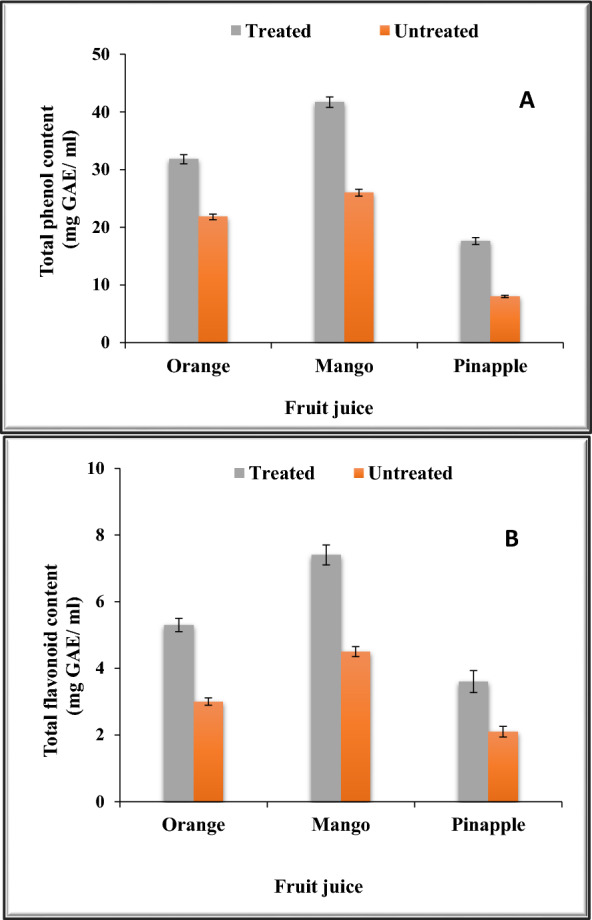
Total phenols (**A**) and total flavonoids (**B**) in treated and untreated fruit juices with chitosan-immobilized pectinase

The results in Fig. 9a show that mango juice had the greatest total phenolic concentration, whereas pineapple juice had the least phenolic content. These results were similar to those of previous studies on pectinase-treated papaya juice [76].

Moreover, apricot juice’s polyphenol content rose after pectinase treatment [8]. Enzymatic hydrolysis may have contributed to the rise in total phenolic content by promoting the activity of cellulases, pectinases, and pectine sterases while also aiding in the inactivation of lipoxygenase, peroxidase, and polyphenol oxidase [38].

Total flavonoids, one of the antioxidant chemicals, are said to be abundant in fruits [19]. Flavonoids are the most familiar and most significant group of phenolic compounds in plants. The crucial chemical structure of flavonoids is a skeleton of diphenylpropane (C_6_C_3_C_6_) [29].

Figure 9b showed the total flavonoid content of the various treated and untreated fruit juices using chitosan-immobilized pectinase. A significant increase in the total flavonoid content after pectinase treatment was associated with increased tissue breakdown and flavonoid release from the peel cell wall. These results are similar to those of a previous study that effectively preserved the juice’s total flavonoid content by treating papaya juice with pectinase [76].

### Antioxidant activity of fruits juice treated with chitosan-immobilized pectinase

**Fig. 10:**
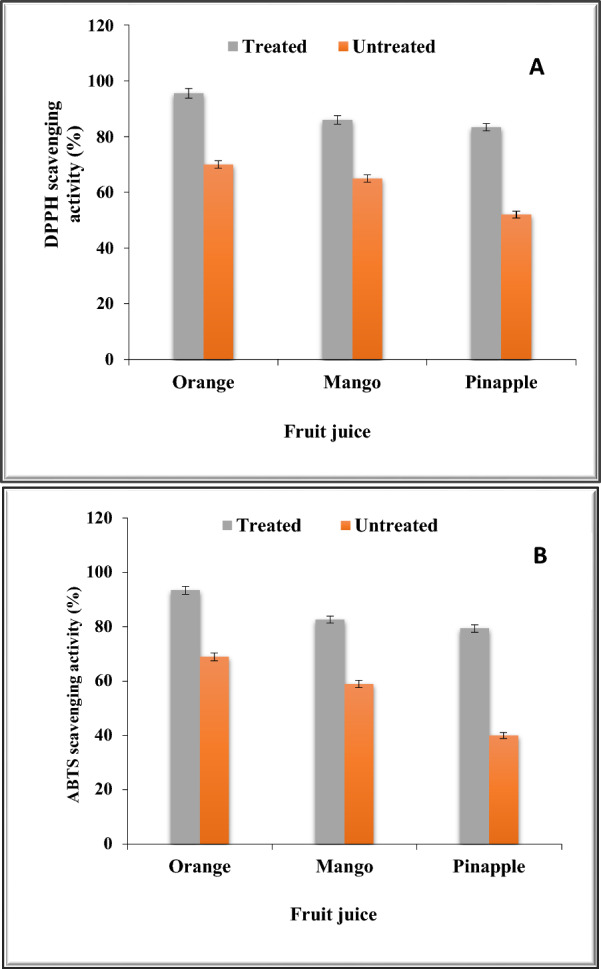
DPPH (**A**) and ABTS (**B**) scavenging activities (%) of the fruit juices treated and untreated with chitosan-immobilized pectinase

The fruits under investigation are rich in total phenols, which may reflect the antioxidant activity which was tested by DPPH (Fig. 10a) and ABTS (Fig. 10b). Fruit juice’s antioxidant activity increased following treatment with chitosan-immobilized pectinase. When compared to the similar untreated juice with pectinase, the fruit juice treated with pure pectinase exhibited a much better ability for radical scavenging. The process of scavenging DPPH is dependent on the antioxidants in fruits’ capacity to donate hydrogen, which results in the creation of non-radical DPPH-H. However, ABTS is implicated in an electron transfer route that transforms ABTS^+^ into ABTS [40].

According to Bashir et al. [8], a rise in antioxidants (phenolics and flavonoids) may be the cause of this increase in scavenging ability. Additionally, it was shown that pectinase encouraged the breakdown of the cell wall, releasing flavonoids and polyphenols that are confined within the cells [68].

While the electron-donating capacity of phenolics appears to be associated with extended electric delocalization throughout the entire molecule [67], the hydrogen-donating capacity of phenolics to scavenge free radical can be defined by the dissociation energy of the bond of OH bond [62]. Hansen and Laroze [36] found similar results with raspberry and apricot juice. Therefore, phenolics and flavonoids are responsible for the fruit juice’s antioxidant potential, and they may work in concert to stop free radicals from damaging biological macromolecules.

## Conclusion

The catalytically proficient pectinase for pectin hydrolysis was purified and immobilized from *Aspergillus nidulans.* Thus, the results indicate the possibility to use *Aspergillus nidulans* for the production of pectinase. The homogeneity of the isolated enzyme was shown by a single band on SDS-PAGE for pure pectinase with a molecular weight of 50 kDa. The purified pectinase was immobilized by entrapment in alginate and cross-linking on chitosan. The possibility of reusing the chitosan and pectinase enzymes that were immobilized in alginate was examined. After the seventh cycle, the immobilized pectinase retained 20.3 and 38.8% on alginate and chitosan, respectively. After 30 days, the immobilized pectinase showed higher activity on chitosan (39.8%) or alginate (17.3%) than the free enzyme (5%). The immobilized pectinase probably showed more stability because immobilization stops autolysis. The fact that the bead creates an environment that supports the growth of the enzyme could be one reason for the immobilized pectinase’s endurance. Chitosan-immobilized pectinase displayed a potential role in clarification of orange, mango and pineapple juices and could be used in food industry to increase juice quality. The chitosan-immobilized purified pectinase exhibited high clarification (97.8, 75.7 and 84.5%) expressed as juice clarification (%) than the crude enzyme (82.4, 61.4 and 70.6%), soluble purified (90, 65.3 and 75.5%) and the control samples (66.7, 55.6 and 64.8%) for orange, mango and pineapple juices. Increased tissue breakdown and flavonoid release from the peel cell wall were linked to a notable rise in the overall flavonoid concentration following pectinase treatment. After chitosan-immobilized pectinase treatment, fruit juice’s antioxidant activity improved. The fruit juice treated with pure pectinase had a much higher capacity for radical scavenging in comparison to the comparable untreated juice with pectinase. The findings support the pectinase immobilization’s economic and industrial benefits, especially with regard to reusability, which increases the likelihood that it will be used in a variety of industrial applications.

## Data Availability

No datasets were generated or analysed during the current study.
